# Mitigation of the Risk of Importing Akabane and Exotic Bluetongue Virus in Ruminant-Sourced Sera

**DOI:** 10.3390/v18091039

**Published:** 2026-09-19

**Authors:** Lisa Orfe, Raymond W. Nims

**Affiliations:** 1 VMRD, Inc., Pullman, WA 99163, USA; 2 Syner·G Biopharma, Boulder, CO 80301, USA

**Keywords:** Akabane fever, Akabane virus, arbovirus, fetal bovine serum, exotic bluetongue virus

## Abstract

Akabane virus is an arbovirus of the *Bunyaviridae* family that is endemic in certain tropical and subtropical regions, including parts of Asia, Africa, the Middle East, and Australia. Since Australian-sourced fetal bovine serum (FBS) is a preferred reagent for use in regulated cell culture applications (including but not limited to manufacturing processes for human and veterinary biologics), the importation of FBS from Australia represents a risk to United States livestock in the form of possible introduction of Akabane virus. This risk is currently mitigated, per United States Department of Agriculture (USDA) mandate, through importation testing and/or barrier treatment of the incoming serum by gamma irradiation at ≥30 kGy. Gamma irradiation, while effective for inactivating any infectious Akabane virus that might be present in the serum pools or finished serum product, is not a practical solution for all types of imported serum products. The testing that is currently required by the USDA for Akabane virus and exotic bluetongue viruses involves the use of virus-naïve sheep, is not characterized (validated) for limit of detection, and is not high-throughput, resulting in delays in satisfying import requirements for importation of serum. This opinion paper discusses these topics in detail and proposes alternative testing methodologies that should overcome the various limitations of the current testing protocol. Following development and validation of the proposed methods for limit of detection at the ultimate USDA testing site, decisions regarding the suitability of these methods to replace the current sheep antibody production assay may be made by the USDA.

## 1. Introduction

Akabane virus is an arbovirus of the *Bunyaviridae* family that is endemic in certain tropical and subtropical regions, including parts of Asia, Africa, the Middle East, and Australia [1]. The virus causes Akabane fever, with symptoms including teratogenesis, fetal death, and abortion in cattle, sheep, and goats. In this regard, the effects of Akabane virus are similar [2] to those caused by the bunyaviruses Cache Valley virus (endemic in the United States) and Schmallenberg virus (endemic primarily in Europe but also reported in Asia and Africa [3]). The insect vectors for Akabane virus include midges of the *Culicoides* genus and mosquito species including *Aedes vexans*, *Culex tritaeniorhyncus*, and *Anopheles funestus* [2]. Akabane fever is not currently endemic in the United States, but there is regulatory concern over importation of this virus in contaminated bovine-sourced raw materials, as “livestock in the United States are considered to be susceptible to Akabane, and indigenous arthropod species may be competent to transmit the virus” [2]. Of special concern is that Australia is a favored geographical region of origin for fetal bovine serum (FBS) from a viral safety and transmissible spongiform encephalopathy (TSE) point of view [4], leading to the sometimes-questioned [5] practice of sourcing FBS preferentially from Australia, New Zealand, and the United States.

What approaches are available to ensure that infectious Akabane virus is not imported as a contaminant of bovine (fetal, newborn and neonatal, calf, and adult) or other ruminant sera (goat and sheep), and which approaches are expected to be most effective? The purpose of this *Opinion* article is to discuss these questions and formulate an optimal strategy, going forward, for preventing importation of this virus and the associated Akabane fever into the United States. A discussion of exotic bluetongue virus testing is included, as the USDA also utilizes the sheep antibody production test to screen quarantined imported ruminant serum for these *Reoviridae* viruses prior to release.

## 2. Bluetongue and Akabane Virus Cross the Placental Barrier

Bluetongue virus (BTV) and Akabane virus are established teratogens in cattle, sheep, and goats, characterized by their capacity to readily breach the placental barrier [6,7,8,9,10,11]. Consequently, FBS and other ruminant-derived blood products sourced from endemic regions carry an inherent risk of infectious viral contamination. Following exposure, the onset of viremia occurs rapidly, typically developing 1 to 6 days post-infection for Akabane virus [8] and 2 to 4 days for BTV [12,13]. While there is a distinct lack of published data quantifying specific circulating viral titers in the dam or fetus for Akabane virus, BTV viremia is extensively documented. Although contemporary BTV viral load determination relies predominantly on molecular methods, a foundational transmission and pathogenesis study utilizing live-virus isolation demonstrated that peak BTV viremia ranges from 10^3.8^ to 10^7.4^ ELD (embryo lethal dose)_50_/mL in whole blood [14].

## 3. Current Importation Risk Mitigation Strategy

The United States Department of Agriculture (USDA) currently mitigates the risk of importing infectious Akabane virus and exotic bluetongue virus (family *Reoviridae*) in ruminant sera from Australia and importing exotic bluetongue virus in ruminant sera from Central America [15] by requiring quarantine of sera imported from these regions until one of the following two options has been satisfied: 1) viral safety testing or 2) gamma irradiation at ≥30 kGy. Sera may currently be imported from Canada and New Zealand directly without satisfying these options. Ruminant sera here are defined as FBS, newborn and neonatal calf serum, calf serum, adult bovine serum, and goat or sheep serum. The two options are further discussed below.

### 3.1. Viral Safety Testing

**Figure 1:**
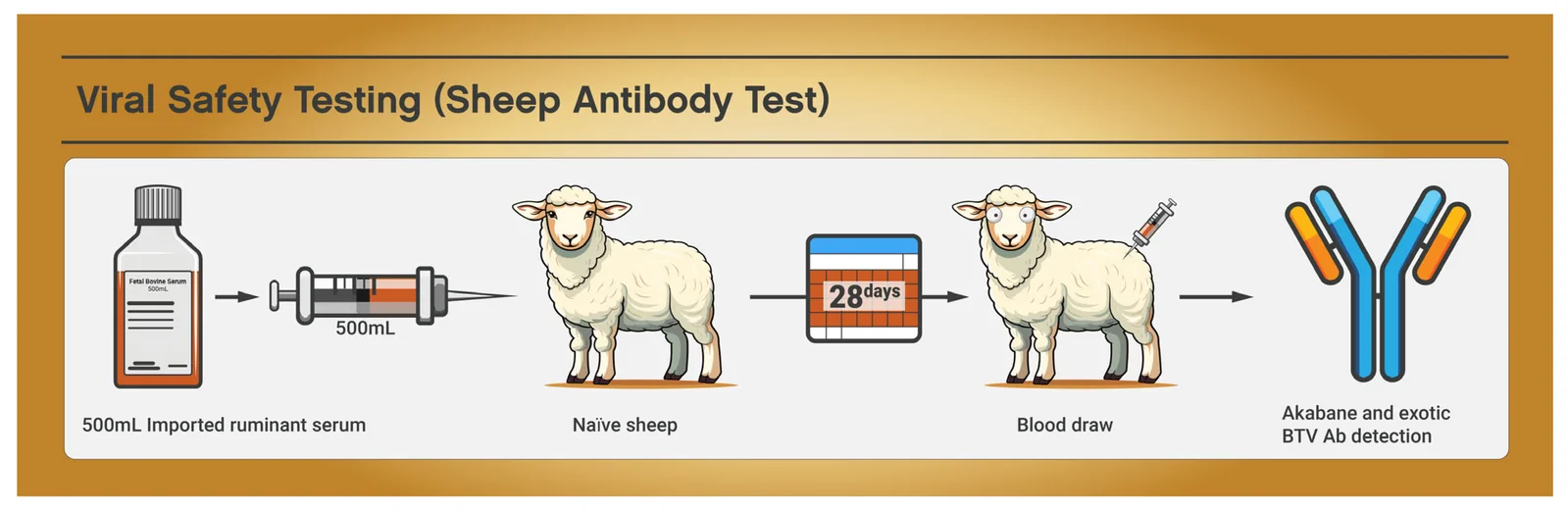
Schematic of the current sheep antibody test used to release imported ruminant sera from certain geographic regions. Abbreviations used: Ab, antibody; BTV, exotic bluetongue virus.

Safety testing [15] involves a 28-day antibody production (seroconversion) test in sheep (Figure 1). It is conducted by the USDA’s National Veterinary Services Laboratory (NVSL). The test sample consists of 500 mL of serum to be collected from every 500 L of serum imported. Serological tests are performed on the sheep prior to inoculation and 28 days following inoculation with the test sample. Seroconversion to antibody positivity (i.e., to Akabane or exotic bluetongue) is indicative of the presence of Akabane or exotic bluetongue antigen in the serum sample under evaluation. A negative result allows the test serum to be released from quarantine, while a positive result leads to destruction of the imported serum lot or shipment back to the country of origin [15].

### 3.2. Gamma Irradiation

The entire volume of the imported serum must be subjected to at least 3 megarads (30 kGy) of gamma radiation in a United States irradiation facility that has been inspected by the USDA and has executed a compliance agreement with the USDA. Irradiation certificates are to be retained for at least two years and must be made available for USDA inspection [15].

## 4. Discussion of the Effectiveness and Limitations of Current Risk Mitigation Approaches

The viral safety testing option mentioned in the USDA Veterinary Services Notice 98-05 [15] has limitations. These can be summarized as (1) uncharacterized sensitivity for detection of Akabane or exotic bluetongue virus; (2) inability to enable quantification of biosecurity risk; (3) lack of adherence to the 3R (replacement, reduction, and refinement of animals used in research, teaching, testing, and exhibition) policies mandating reductions in the use of animals for research purposes [16]; and (4) logistical limitations leading to delays in initiating and completing the required testing.

### 4.1. Uncharacterized Detection Sensitivity

Institutional Animal Care and Use Committees (IACUCs) generally prohibit the use of animals for validation purposes. Because of this, the current testing protocol involving antibody production in virus-naïve sheep has not been validated for the limit of Akabane virus or exotic bluetongue virus detection through the types of virus spike–recovery studies that would be expected of an analytical limit method for detecting impurities per the United States Pharmacopeia (USP) <1225> [17] and the International Conference for Harmonisation (ICH) Q2 (R2) [18].

### 4.2. Inability to Enable Quantification of Biosecurity Risk

Protecting United States agricultural livestock from exotic pathogens requires a rigorous, data-driven approach to risk assessment. Because the in vivo sheep assay inherently lacks a definable limit of detection, as noted above, true risk cannot be quantitatively assessed. A detection sensitivity threshold that remains a blind spot cannot be managed. Furthermore, a systematic evaluation of the detection capabilities of in vivo vs. in vitro adventitious virus assays [19] demonstrated that animal models may fail to detect viruses detected by their in vitro counterparts, often requiring viral concentrations multiple log_10_ higher than those required by cell culture methods to trigger a positive detection event. Transitioning to an optimized in vitro platform should replace this unquantifiable biological uncertainty with the exact analytical precision required for the USDA to accurately assess risk to United States livestock that could be introduced through the importation of foreign ruminant serum.

### 4.3. Lack of Adherence to the 3Rs

Recent revamping of ICH Q5A (R2) [20] has replaced most animal-based testing methods used to establish the adventitious viral safety of human biologics. Thus, the in vivo virus screening assay, which utilized suckling and adult mice, guinea pigs, and hen’s eggs, and animal (rat, mouse, and hamster) antibody production assays are no longer mandated. These animal tests have been replaced with nucleic acid-based technologies. Like the sheep antibody production assay mandated by the USDA, ICH-mandated animal-based methods were considered unvalidatable regarding the limit of detection for the viral impurities being tested. Taking these factors into consideration, it becomes hard to justify the continued use of animals in a viral safety test that cannot be characterized for detection limit.

### 4.4. Logistical Limitations Causing Testing Delays

A stakeholder alert, Delays in Testing and Release of Quarantined Imported Fetal Bovine Serum, was sent from the USDA Animal and Plant Health Inspection Service (APHIS) on June 17, 2025, informing stakeholders that “Since February 2025, there have been delays in this safety testing due to a lack of suitable sheep for the bioassay. Samples are in the queue for testing in the order that they are received at NVSL. Efforts continue to procure sheep nationwide and testing continues for the imported FBS as quickly as possible as suitable sheep are available. APHIS expects these challenges will continue and is discussing with serum industry representatives the options for safety testing and mitigation treatments to replace the sheep bioassay.” The primary challenge in procurement is identifying sheep that are seronegative for BTV.

### 4.5. Efficacy of Gamma Irradiation at ≥30 kGy for Akabane and Other Simbu Serogroup Bunyaviruses

The gamma irradiation option also has certain limitations. These include primarily practical considerations, while inactivation efficacy and, therefore, effectiveness for achieving the desired importation prevention goals are not disputed.

The literature on the efficacy of gamma irradiation for inactivating viruses in bovine serum has been reviewed [21]. The challenge viruses that have been evaluated include the Simbu serogroup bunyaviruses Aino and Akabane [22] and Cache Valley virus [23]. Each of these viruses was readily inactivated by gamma irradiation, with log_10_ reductions per kGy of 0.29, 0.40 and ≥0.159, respectively. This suggests that a 30-kGy fluence will result in 8.7, 12, and ≥4.8 log_10_ inactivation of the three bunyaviruses, respectively.

### 4.6. Efficacy of Gamma Irradiation at 30 kGy for Exotic Bluetongue Viruses

Data on the inactivation of exotic bluetongue virus variants do not exist in the literature reviewed [21]. House et al. [22] published data on bluetongue virus, from which a log_10_ inactivation per kGy of 0.12 has been calculated [21]. This corresponds to 3.6 log_10_ inactivation for a 30-kGy fluence. Additional data on bluetongue virus [21] indicate efficacies of 0.11 to 0.15 log_10_ inactivation per kGy, corresponding to 3.3 to 4.5 log_10_ inactivation for a 30-kGy gamma radiation fluence.

### 4.7. Practical Limitations of the Gamma Irradiation Option

Gamma irradiation or other viral inactivating treatment is mandated for FBS to be used in biologics manufacturing. “The use of non-inactivated serum should be justified” per the European Medicines Agency [24]. In European Pharmacopoeia monograph 2262 [25], the following statement addresses the expectation for viral inactivation treatment: “For bovine serum intended for use in immunological veterinary medicinal products, for inactivation by gamma irradiation a minimum dose of 30 kGy is applied, unless otherwise justified and authorised.” However, basic research (especially) and even some biologics manufacturing processes that employ ruminant serum subject to the USDA testing requirement may be adversely impacted by the changes in serum performance resulting from gamma irradiation. The key consideration for determining the suitability of gamma-irradiated serum is the potential impact of irradiation on serum performance for the individual cell culture or research application under evaluation. This determination must be made on a case-by-case basis for each application, typically by the end users and not by the serum supplier. Therefore, routine gamma irradiation of all imported serum pools and finished serum products is not a realistic option. As a result, the current USDA serum testing paradigm requires replacement rather than elimination.

## 5. Recommendation for Development and Validation of Methods to Replace the USDA Sheep Safety Test

The 9 CFR 113.53 [26] assay format that is currently being used to test animal-derived materials for species-specific viruses of concern is proposed as the best alternative to replace the USDA sheep antibody production test for Akabane virus and exotic bluetongue viruses.

**Figure 2:**
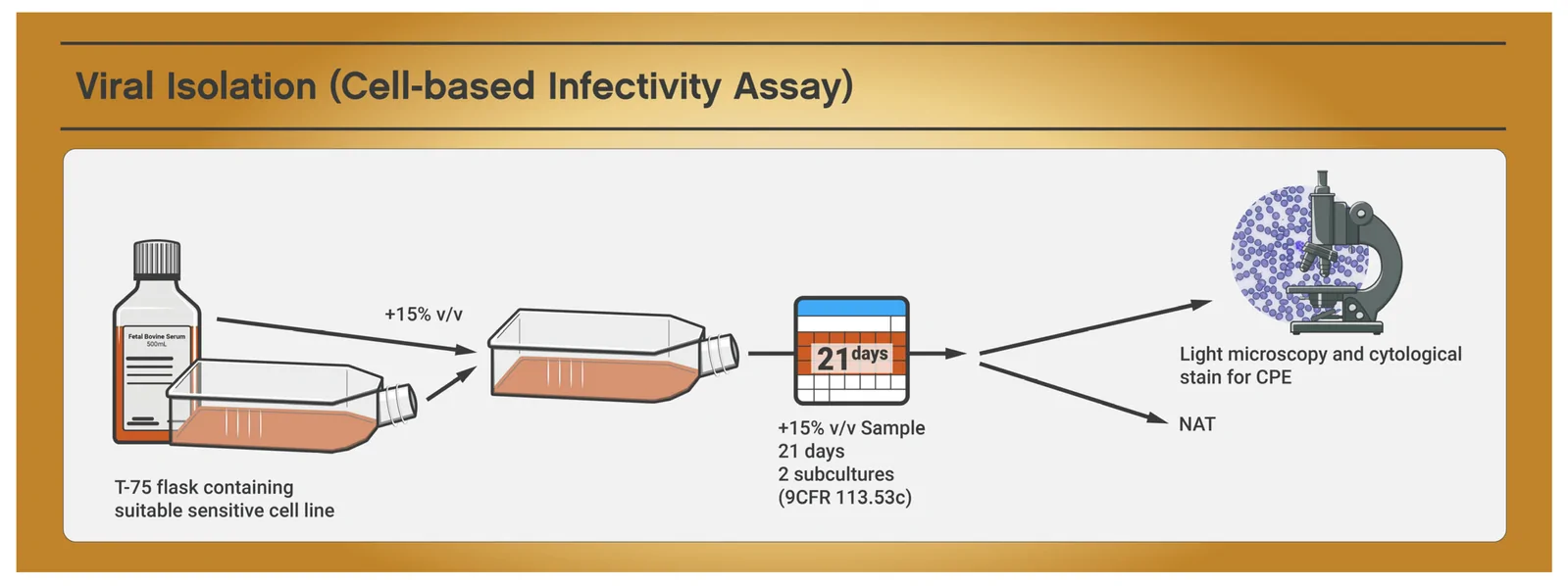
Schematic of the proposed replacement methods. Abbreviations used: CPE, viral cytopathic effect; NAT, nucleic acid testing; *V*/*V*, volume to volume.

Testing for Akabane virus. The authors propose a modified 9 CFR 113.53 method that follows the same protocol as the test currently performed for bovine serum (Figure 2), except that the bovine detector cell would be replaced with the BHK-21 cell. The BHK-21 cell line is known to have broad applicability for the detection of arboviruses per USP <1437> [27] and, in fact, has been characterized as susceptible to infection by Akabane virus [8]. The Vero detector cell line that is typically used in the 9 CFR 113.53 method is also known to be permissive to Akabane virus infection with the generation of cytopathic effects [28] and therefore should be retained. Simbu group bunyaviruses (exemplified by Cache Valley virus) display rapid amplification in cell culture, causing rapid deterioration of cell monolayers as an easily identified viral cytopathic effect [29]. Thus, the proposed cell-based infectivity assay would integrate a relatively high test-serum inoculation level (15% *v*/*v* over the 21-day assay) with the enhanced detection sensitivity conferred by multiple (at least two) cellular passages (Figure 2). The assay sensitivity should be further enhanced by the expected rapid and easily detected cytopathic effect endpoint (bunyaviruses are not expected to cause hemadsorption of erythrocytes). The method may be characterized for limit of Akabane virus detection using standard viral spike–recovery validation protocols (see the Supplemental Materials for a high-level workflow that might be followed during method validation). The 9 CFR 113.53 assay design [26] incorporates a fluorescent antibody staining step to identify specific targeted extraneous viruses, and in certain cases (i.e., absence of cytopathic effect and hemadsorbing ability), it may be the only endpoint applicable for a specific virus of concern (such as non-cytopathic bovine viral diarrhea virus). For the proposed Akabane cell culture assay, an RT-qPCR endpoint is suggested for use [30] at the conclusion of the 21-day maintenance period. This nucleic acid-based test serves two critical functions: First, it confirms that any observed cytopathic effect is due to Akabane virus and not some other Simbu group bunyavirus (e.g., Cache Valley virus or Schmallenberg virus) or other unrelated adventitious virus. Second, this endpoint enables the detection of non-cytopathic strains of the target virus (just as the current 9 CFR 113.53 method detects non-cytopathic strains of BVDV), if such exist.

Testing for exotic bluetongue virus. We see no reason why the current 9 CFR 113.53 method [26], which uses Vero cells and a bovine detector cell, such as bovine turbinate (BT) cells (Figure 2), should not be suitable for detecting exotic bluetongue viruses. Reoviruses such as bluetongue virus are expected to cause cytopathic effects, and the same considerations for establishing assay sensitivity (limit of detection) discussed above for Akabane should apply to the use of the proposed method to detect exotic bluetongue viruses. Consistent with the proposed Akabane method, a nucleic acid-based test should be employed at the conclusion of the 21-day maintenance period. This endpoint confirms that any positive cytopathic effect result obtained in a routine assay is due to exotic bluetongue virus, ruling out endemic bluetongue virus or other adventitious virus (Figure 2). Additionally, this endpoint enables the detection of any non-cytopathic strain of exotic bluetongue virus, if such exists.

## 6. Conclusions

The USDA has the critical mandate of protecting the United States agricultural economy and serves as the ultimate gatekeeper against the introduction of foreign animal diseases, including Akabane virus and exotic bluetongue viruses, via imported ruminant serum. To best support this mission, the legacy in vivo sheep antibody production test should be replaced. The fundamental flaw in the current testing paradigm lies in its inability to be characterized for a limit of detection. Without a known detection threshold, quantifying the true risk to United States livestock is scientifically impossible. We advocate for the development by the USDA of highly sensitive in vitro (cell-based) methodologies that can be rigorously validated for limit of detection. By adapting currently existing USDA-approved cell-based infectivity platforms and incorporating nucleic acid-based identity confirmation approaches, the USDA can be equipped with a modernized, analytically robust tool to actively quantify and mitigate risks to United States livestock.

## 7. Limitations

The authors, while being subject matter experts in viral testing of biological samples (including the 9 CFR 113.53 method as applied to animal sera), are unable to conduct the empirical studies needed to confirm the suitability of the proposed methods for detecting the presence of infectious Akabane virus or exotic bluetongue virus in a ruminant serum matrix. As USDA Select Agents, infectious Akabane virus and exotic bluetongue virus stock possession and use is strictly restricted within the United States. To our knowledge, only the NVSL or other USDA sites may have access to such virus stocks. As the NVSL or another USDA site will be the ultimate testing facility for any methods used to replace the sheep antibody production test, development of the proposed replacement methods (confirmation of cytopathic effect in the proposed detector cell lines; development of a nucleic acid-based method to confirm the identity of a virus isolate generated in the proposed assays, and validation of the final methods for limit of detection of the specific viruses targeted) will necessarily need to be completed by the USDA at the ultimate testing site. A high-level experimental design for such a validation exercise is provided in the Supplemental Materials.

## Data Availability

No new data were generated in the course of this work.

## Supplementary Materials

The following supporting information can be downloaded at: https://www.mdpi.com/article/10.3390/v18091039/s1, A high-level experimental design for validation of the proposed methods is provided in the Supplemental Materials.
