# Supplementary material for: Mitigation of the Risk of Importing Akabane and Exotic Bluetongue Virus in Ruminant-Sourced Sera
Source: Viruses. 2026 Sep 19;18(9):1039. doi: 10.3390/v18091039 (PMC13612089; doi:10.3390/v18091039)
Supplement: Supplementary file 1 [file viruses-18-01039-s001.zip › viruses-4537052-supplementary.pdf]

Mitigation of Risk of Importation of Akabane and Exotic Bluetongue Virus in Ruminant-Sourced Sera

Lisa Orfe and Raymond W. Nims

A Suggested Validation Strategy

1. **Cell Line Qualification and Viral Standardization** To ensure the integrity of the 21-day cell maintenance cycle, it is recommended that baseline growth kinetics, cellular morphology, and maximum passage limits first be established for both the BHK-21 and Vero cell lines (Akabane virus method) and BT and Vero cells (exotic bluetongue virus method). Prior to matrix challenge, reference viral stocks should be titrated to establish standard infectious doses ($TCID_{50}$/mL) utilizing the respective detector cell lines. We recommend using reference stocks of the target viral entities (i.e., Akabane virus and an exotic bluetongue virus) rather than surrogate strains, such as a Simbu group bunyavirus or a surrogate bluetongue virus. Results obtained from surrogate viruses may not reliably extrapolate to the target viruses.
2. **System Suitability Testing (SST)** In accordance with ICH Q2(R2), system suitability criteria need to be defined to establish assay validity. For example, each analytical run could incorporate a concurrent negative matrix control (15% v/v negative bovine serum) and a positive viral control spiked at 2-3× the established Detection Limit (DL). Run acceptance would dictate that the negative control remains strictly free of viral CPE and RT-qPCR amplification, while the positive control must yield viral CPE and positive molecular confirmation.
3. **Matrix Cytotoxicity and 9 CFR 113.53 Compliance** To evaluate potential matrix-induced cytotoxicity that could confound the interpretation of cytopathic effect (CPE), a mock-infection validation phase needs to be conducted in accordance with 9 CFR 113.53 guidelines. Monolayers of both BHK-21 and Vero cells (Akabane virus method) or BT and Vero cells (exotic bluetongue virus method) may be inoculated with confirmed negative bovine serum at a concentration of 15% v/v in the culture medium. Cultures may then be maintained for a contiguous 21-day period, incorporating a minimum of two subcultures. Cellular viability and monolayer confluence should be monitored longitudinally to confirm that the 15% v/v matrix does not induce monolayer degradation or pseudo-CPE (cytopathic effects unrelated to virus).
4. **Analytical Sensitivity (Detection Limit)** In alignment with United States Pharmacopeia (USP) chapter <1225> guidelines for a Category II Limit Test, the validation of this qualitative method will focus primarily on establishing rigorous Specificity and the analytical Detection Limit (DL), exceeding these requirements by also evaluating Intermediate Precision and Robustness as outlined in ICH Q2(R2). The detection limit (DL) of the cell culture systems may be determined via virus spike-and-recovery testing. Confirmed virus-negative bovine serum may be spiked with serial dilutions of Akabane virus or exotic BTV. The spiked matrices (15% v/v) may then be applied to the appropriate detector cells (BHK-21 and Vero for Akabane, and BT and Vero for exotic BTV), and subjected to the complete 21-day, two-subculture maintenance protocol. The DL is defined as the lowest viral titer capable of producing consistent, reproducible viral CPE at the termination of the final subculture.

5. **Analytical Specificity and Nucleic Acid Confirmation** To definitively differentiate true viral replication from non-specific matrix artifacts or infections by non-target viruses, a molecular confirmation strategy is needed. Upon observation of CPE at the final subculture (or at 21 days post-inoculation), nucleic acids may be extracted from the culture supernatant and cellular lysates. The presence of the target virus may then be confirmed via validated, target virus-specific reverse transcription quantitative PCR (RT-qPCR). Furthermore, analytical specificity should be evaluated by challenging the BHK-21/Vero system with endemic adventitious bovine viruses (e.g., cytopathic bovine viral diarrhea virus (BVDV) and bovine parainfluenza virus type 3 (BPIV3)) and the BT/Vero system with endemic (non-exotic) BTV serotypes. Because these endemic viruses are fully capable of inducing viral CPE in these detector cell lines, specificity in this assay is defined entirely by the molecular readout. In a routine assay, the presence of adventitious viral replication and consequent monolayer degradation must be confirmed using RT-qPCR for Akabane virus or exotic BTV for a positive result to be assigned. It follows then that the lowest dilution of virus causing both CPE and positive nucleic acid testing results should be assigned as the DL. In addition, it is possible that the proposed methods could detect non-cytopathic strains of the target viruses (just as the current 9 CFR 113.53 detects non-cytopathic strains of BVDV). The inclusion of the nucleic acid endpoint therefore enables the detection of non-cytopathic strains of Akabane, if such exist.
6. **Precision (Repeatability and Intermediate Precision**) Intra-assay and inter-assay precision should be evaluated to ensure operational consistency under standard conditions. Repeatability (intra-assay precision) may be established by evaluating multiple replicates of serum spiked at the defined DL within a single continuous run. Intermediate precision may be validated by executing the 21-day protocol on DL-spiked samples across independent operational variables, including staggered initiation days and disparate detector cell passage numbers. Intermediate precision based on analysts is generally not useful for a 21-day assay in which multiple analysts will likely perform different steps (cell seeding, sample inoculation, refeeding, subculture, CPE scoring, and RT-qPCR)
7. **Robustness** To satisfy ICH Q2(R2) robustness expectations, the methods may be subjected to deliberate, minor variations in method parameters to verify reliability in a standard laboratory environment. DL-spiked samples may be evaluated under different conditions, including altered serum concentrations (e.g., 14% and 16% v/v), slight deviations in incubator temperature (±1°C), and variations in the timing of the mandatory subcultures (±24 hours). The assay is considered robust if these deliberate perturbations do not result in false negatives or false positives at the final molecular confirmation step.

Table S1: Suggested Assay Validation Parameters and Acceptance Criteria

Validation Parameter	Target Objective	Proposed Test Matrix & Conditions	Recommended Acceptance Criteria
**System Suitability Testing (SST)**	Establish assay validity for each analytical batch.	Concurrent negative matrix control (15% v/v serum) and positive viral control (2-3× DL).	Negative control remains viral CPE/RT-qPCR negative; positive control yields definitive viral CPE and positive molecular confirmation.
**Matrix Cytotoxicity** *(Specificity)*	Confirm 15% v/v serum tolerability over the 21-day maintenance cycle.	Negative bovine serum (15% v/v) on BHK-21, BT, and Vero cell lines.	Maintenance of >90% cellular viability at day 21 (≥2 subcultures); absence of matrix-induced pseudo-CPE.
**Detection Limit (DL)** *(Sensitivity)*	Establish the lowest viral titer capable of reliable qualitative detection.	Serial dilutions of Akabane virus (BHK-21 and Vero cells) and exotic BTV (BT and Vero cells) in 15% v/v serum.	100% detection of viral CPE confirmed by positive RT-qPCR at the defined DL threshold across all replicates.
**Analytical Specificity** *(Exclusivity)*	Demonstrate the assay does not cross-react with non-target viruses.	Serum spiked with endemic adventitious agents (e.g., BVDV, BPIV3) or endemic BTV.	Non-target virus CPE may be observed; however, confirmatory RT-qPCR for Akabane virus or exotic BTV must remain definitively negative.
**Precision** *(Repeatability & Intermediate)*	Establish intra-assay and inter-assay operational consistency.	DL-spiked serum tested in concurrent replicates, and across different days and detector cell passages.	100% qualitative agreement (CPE and RT-qPCR positive) across all replicates and independent operational variables.
**Robustness**	Verify assay reliability against deliberate, minor method perturbations.	DL-spiked samples subjected to altered serum concentrations (14/16%), incubation temperature (±1 °C), and subculture timing (±24 hours).	Deliberate method perturbations do not result in false negatives or false positives at the final molecular confirmation step.
